# Access to medicines and hepatitis C in Africa: can tiered pricing and voluntary licencing assure universal access, health equity and fairness?

**DOI:** 10.1186/s12992-017-0297-6

**Published:** 2017-09-13

**Authors:** Yibeltal Assefa, Peter S. Hill, Anar Ulikpan, Owain D. Williams

**Affiliations:** 1 School of Public Health, the University of Queensland, Brisbane, Australia; 2 Abt Associates, Brisbane, Australia

**Keywords:** Hepatitis C, Access to medicine, Tiered pricing, Voluntary licensing, Universal access, Health equity, Africa

## Abstract

**Background:**

The recent introduction of Direct Acting Antivirals (DAAs) for treating Hepatitis C Virus (HCV) can significantly assist in the world reaching the international target of elimination by 2030. Yet, the challenge facing many individuals and countries today lies with their ability to access these treatments due to their relatively high prices. Gilead Sciences applies differential pricing and licensing strategies arguing that this provides fairer and more equitable access to these life-saving medicines. This paper analyses the implications of Gilead’s tiered pricing and voluntary licencing strategy for access to the DAAs.

**Methods:**

We examined seven countries in Africa (Egypt, Ethiopia, Nigeria, Democratic Republic of Congo, Cameroon, Rwanda and South Africa) to assess their financial capacity to provide DAAs for the treatment of HCV under present voluntary licensing and tiered-pricing arrangements. These countries have been selected to explore the experience of countries with a range of different burdens of HCV and shared eligibility for supply by licensed generic producers or from discounted Gilead prices.

**Results:**

The cost of 12-weeks of generic DAA varies from $684 per patient treated in Egypt to $750 per patient treated in other countries. These countries can also procure the same DAA for 12-weeks of treatment from the originator, Gilead, at a cost of $1200 per patient. The current prices of DAAs (both from generic and originator manufacturers) are much more than the median annual income per capita and the annual health budget of most of these countries. If governments alone were to bear the costs of universal treatment coverage, then the required additional health expenditure from present rates would range from a 4% increase in South Africa to a staggering 403% in Cameroon.

**Conclusion:**

The current arrangements for increasing access to DAAs, towards elimination of HCV, are facing challenges that would require increases in expenditure that are either too burdensome to governments or potentially so to individuals and families. Countries need to implement the flexibilities in the Doha Declaration on Trade Related Intellectual Property Rights agreement, including compulsory licensing and patent opposition. This also requires political commitment, financial will, global solidarity and civil society activism.

## Background

It is estimated that a total of 115 million people are infected with hepatitis C virus (HCV) globally. Around 80 million of these people are living with chronic infection and millions more are newly infected each year. Annually, 700,000 people die from HCV-related complications, including cirrhosis and liver cancer [1]. Close to 15% of the people with HCV infection live in high income countries (HICs), 73% live in middle income countries (MICs), and 12% in low-income countries (LICs). Despite the overall high global burden of the HCV epidemic, the capacity to respond has been very limited [2].

This inadequate response can in part be explained by the fact that there have, until recently, been relatively poor rates of cure from the infection or its complications. The previously available treatment for HCV, Pegylated interferon and ribavirin, involved a prolonged regimen of 24–36 weeks that was expensive, poorly tolerated, and had only 54%–63% rates of efficacy. Moreover, this treatment was often unavailable to people living in low- and middle-income countries (LMICs) where most of those infected with HCV reside [3].

However, the recent introduction of the Sofosbuvir (Sovaldi®) and Harvoni (Sofosbuvir/Ledipasvir) as key Direct Acting Antivirals (DAA) promises a more rewarding future for treating HCV as these drugs can cure 90–95% of the cases within 8–12 weeks, and thereby significantly reduce the risk of developing cirrhosis and liver cancer [4]. DAAs offer a range of advantages compared with the interferon based drugs: there are fewer side effects and higher cure rates, including for those in advanced stages of infection. As such, there is increasing awareness as to how DAAs could revolutionise the fight against HCV and assist in the world reaching the international target of elimination by 2030, not least if 80% coverage of those infected can be achieved. With an increase in coverage of DAA from present globally low rates, combined with focused strategies for infection control, screening and treatment, there is the real potential to eliminate HCV [5].

Yet the challenge facing many individuals and countries today does not lie with the efficacy of the DAA treatments, but with their ability to access these treatments due to their relatively high prices [6]. High prices for DAAs, inadequate health financing by many LMIC governments, and high rates of out-of-pocket-expenditure (OOPE) on health have combined to produce slow uptake of a revolutionary therapy [7, 8]. This reality persists despite strategies by Gilead Sciences—the pharmaceutical company which owns the patents for the new DAAs — to apply differential pricing and licensing strategies, arguing that this provides fairer and more equitable access to these life-saving medicines. In HICs governments and consumers still pay “standard prices” (although these are presently also falling); in some (but not many) middle-income countries (MICs) “tiered pricing” and voluntary licences are applied and made available. Gilead’s licenses for generic production, particularly to Indian generic companies (11 of these in 2017), have the specific provision that those firms can export to third party low-income countries (LICs) with little or no pharmaceutical manufacturing capacity themselves [9]. We note that these arrangements exclude many MICs from price discounted or licensed generic supply, despite their often high HCV prevalence [10].

This paper analyses the implications of Gilead’s current tiered pricing and voluntary licencing strategy for access to the DAAs. Using a convenience sample of African countries, it models data that questions whether these marketing strategies will resolve a potential shortfall in coverage of DAAs. Our hypothesis is that these instruments and interventions on price and generic supply, while welcome, will fail to achieve the realisable goal of disease elimination and are insufficient for achieving fair and equitable access to DAAs. While other interventions and investments are required in country health systems to eliminate HCV (for example in infection control and screening,) the paper sets out to illustrate that the prices even on ‘discounted’ licensed DAAs places them out of reach of many people with low incomes and from governments with little capacity (or political will) to increase health spending.

## Methods

We examined seven countries in Africa (Egypt, Ethiopia, Nigeria, Democratic Republic of Congo (DRC), Cameroon, Rwanda and South Africa) to assess their financial capacity to provide DAAs for the treatment of HCV under present voluntary licensing and tiered-pricing arrangements. These LMICs have been selected to explore the experience of countries with a range of different burdens of HCV and shared eligibility for supply by licensed generic producers or from discounted Gilead prices, with Egypt constituting an exception whereby unlicensed generics are being manufactured.

The prices used for our modelling were taken from the 2016 World Health Organization report, which provides the most up-to-date prices for DAAs in countries eligible for price discounts either from Gilead (as the originator firm), or prices offered by generic producers of DAAs under license [11]. It should be noted that our analysis does not model on the basis of the ‘actual market prices’ being paid for DAAs in these countries either by governments or individuals, nor does it explore if countries have actually initiated DAA access programs on the basis of the emerging price discounts and licensed generic availability. Moreover, we used conservative estimates: prices for 12-weeks regimens, lowest and factory gate prices, assumed zero re-infection, ignored competing health priorities and complex co-morbidities. Thus the objective was to underscore the disparity between voluntary licensed prices and the somewhat aspirational goal (albeit one that is justifiable on the basis of the right to health) of providing universal coverage of all those living with HCV in the selected countries (or of meeting WHO’s target of 80% coverage). We highlight what appears to be a clear cut for intervening on present prices for DAAs by governments or the global health community, in order to make treatment goals more affordable, and access to DAAs realistic.

From the two sets of prices – the tiered and generic substitute prices – we modelled the extent to which equitable access to DAAs for HCV patients in these LMICs might be achieved. In this assessment, we have used a number of available and widely accepted indices on country/individual income (e.g. Gross Domestic Product (GDP)), alongside total country expenditure on health. The paper then triangulates these admittedly general proxies for the ability to pay for HCV treatment with the burden of disease of HCV in each country [12]. We thus calculated the percentage of the cost of 12-weeks DAA for all HCV patients against the total annual health expenditure in each country. The total health expenditure (THE) was calculated as the sum of government and private expenditures. It did not include overseas development assistance.

The different sources of health financing and their percentage contribution in each country are summarized to show the extent of OOPE, government expenditure and, in addition, overseas development assistance. We estimated the required percentage increases by any of the component sources of financing in order to provide DAAs to all HCV infected patients in each country, and thereby achieve the international target of HCV elimination.

The paper also offers an additional important consideration in judging the ability to pay for medicines, and assesses how country income inequality (and rates of out of pocket expenditure) clearly interacts with the presence or absence of a functioning health insurance mechanism, and the proportions of government expenditure and OOPE in relation to THE. All these variables are also considered in the analysis. The percentage of estimated cost of 12-weeks DAA per HCV patient against the median per capita income was thus estimated. Moreover, we conducted modelling to estimate the cost burden of the 12-weeks DAA per HCV patient with different wealth quintiles.

We used Google, Google Scholar and PubMed to search for data related on total anti-HCV population [13], THE (OOPE, government and development partners) [14], cost of DAA for 12-weeks [11], Gini-coefficient and median per capita income [15] for the seven countries under investigation. We used multiple databases from the World Health Organization, World Bank, Gilead Sciences, and other sources, including peer-reviewed papers and reports on DAAs and HCV. Data analysis was conducted using excel spread sheet.

## Results

The findings of this study assess the affordability of voluntary licensed generics (and Egypt’s unlicensed product) and the originator firm’s tiered prices for DAAs (at 2016 listed prices in USD), and then in terms of a number of methodological steps that employ some major standard international indices for health expenditure and income. Thus price and affordability of voluntary licenced and tiered priced treatments are measured against: (1) total country health expenditure on health calculated as government expenditure plus private expenditure (without overseas development assistance); (2) the required increase in health expenditure by different sources of health financing in each case study country required to eliminate each country burden of HCV (including ODA for health); and, (3) the cost of DAAs in the context of figures expressing national income inequality and rates of OOPE in almost all the countries under investigation. The findings are presented sequentially in four sections with four tables and one figure depicting: (1) burden of HCV infection and the total cost of treating all those infected at 2016 prices; (2) the sources of health financing in each country and their percentage contribution to THE and THE plus ODA for health; (3) the required increase in health expenditure by different sources to achieve HCV elimination by full coverage; and, (4) the context for elimination and access to fair and equitable medicines provide by national income inequality and cost of DAA per patient.

### Burden of hepatitis C virus infection and the total cost of its treatment

Burden of HCV varies greatly across the countries considered in this study. The number of people with HCV infection ranges from 110,000 in DRC to more than 8 million in Egypt and Nigeria. The prevalence of HCV is 14.5% in Egypt, 8.4% in Nigeria, and 11.6% in Cameroon. For the remaining countries, the prevalence of HCV is arguably manageable, and elimination is a realistic public health goal (Table 1).

**Table 1:** Hepatitis C Virus burden, total health expenditure and cost of HCV treatment in seven African countries

Country	Total HCV population (000,000)^a^	Total expenditure on health as % of GDP (2014) (WHO)^b^	Total expenditure on health (000,000) (2014) (WHO)^b^	Cost of 12- weeks regimen of DAA per patient^c^		Total cost of 12- weeks regimen of DAA (000,000)				Cost of 12-weeks DAA as % of total health expenditure			
						For universal (100%) DAA coverage		For 80% DAA coverage		For universal (100%) DAA coverage		For 80% DAA coverage	
				Generic	Originator	Generic	Originator	Generic	Originator	Generic	Originator	Generic	Originator
Egypt	8.306	5.6%	18,524	$684	$1200	$5681.3	$9967.2	$4545.0	$7973.8	31%	54%	25%	43%
Ethiopia	0.676	4.9%	3015	$750	$1200	$507.0	$811.2	$405.6	$649.0	17%	27%	13%	22%
Nigeria	8.115	3.7%	17,800	$750	$1200	$6086.3	$9738.0	$4869.0	$7790.4	34%	55%	27%	44%
DRC	0.11	4.3%	1515	$750	$1200	$82.5	$132.0	$66.0	$105.6	5%	9%	4%	7%
Cameroon	1.473	4.1%	1197	$750	$1200	$1104.8	$1767.6	$883.8	$1414.1	92%	148%	74%	118%
Rwanda	0.475	7.5%	607	$750	$1200	$356.3	$570.0	$285.0	$456.0	59%	94%	47%	75%
South Africa	0.633	8.8%	27,519	$750	$1200	$474.8	$759.6	$379.8	$607.7	2%	3%	1%	2%

^a^[13]

^b^ http://gamapserver.who.int/gho/interactive_charts/health_financing/atlas.html?indicator=i2

^c^ http://apps.who.int/iris/bitstream/10665/250625/1/WHO-HIV-2016.20-eng.pdf?ua=1

The cost of 12-weeks of generic DAA varies from $684 per patient treated in Egypt to $750 per patient treated in other countries. These countries can also procure the same DAA for 12-weeks of treatment from the originator, Gilead, at a cost of $1200 per patient. The prices of generic drugs are close to 40% cheaper than the prices of drugs from the originator (Table 1).

In absolute terms, universal treatment of HCV would be a major expenditure for these countries: in Egypt, the total cost of 12-weeks use of DAA to treat its HCV burden ranges from USD $5.7 billion (from local generic manufacturers) to USD $9.9 billion (from originator); in Nigeria, it costs USD $6 billion (from primarily Indian generic sources) and USD $9.7 billion (from the originator firm). The total cost of generic DAA to achieve universal coverage of all those presently living with HCV ranges from 2% of current THE (in South Africa) to 92% in Cameroon. To achieve the same outcome with originator DAAs increases these projections from 3% of THE in South Africa to 148% of THE in Cameroon (Table 1).

We also estimated the cost of DAAs required to meet the 80% coverage target that WHO recommended as necessary toward elimination. We found that the cost of generic DAAs will be 25%, 13%, 27%, 4%, 74%, 47% and 1% of THE in Egypt, Ethiopia, Nigeria, DRC, Cameroon, Rwanda and South Africa, respectively. This is still a substantial cost to countries and populations though it is obviously less that the cost of DAAs for universal coverage (Table 1).

### Sources of health financing and their percentage contribution

In general, health care in most LMICs is financed by government (Govt) or private (Prvt) expenditures (including insurance and OOPE) sources, and overseas development assistance (ODA), in differing combinations. Table 2 shows that the contribution of each source of financing of THE varies from country to country. The government share of THE is very low in Cameroon (22.9%) and Nigeria (25.1%) as compared to Ethiopia (58.7%) and South Africa (48.2%). The government contribution to health financing is less than the contribution from private sources in all countries except Ethiopia (Table 2).

**Table 2:** Sources of health financing and their percentage contribution in seven African countries, 2014

Country	Total Health Expenditure (THE) = GovtE+PrvtE^a^								Overseas development assistance (ODA)^a^		THE plus ODA
	Government expenditure (GovtE)		Private expenditure (PrvtE)		Out of pocket expenditure (OOPE)		THE				
	N	% of THE	N	% of THE	N	% of PrvtE	N	% of THE+ ODA	N	% of THE + ODA	N
Egypt	7076	38.2%	11,448	61.8%	10,318	90.1%	18,524	98.7%	238	1.3%	18,762
Ethiopia	1770	58.7%	1245	41.3%	974	78.2%	3015	70.6%	1257	29.4%	4272
Nigeria	4468	25.1%	13,332	74.9%	12,763	95.7%	17,800	93.7%	1193	6.3%	18,993
DRC	559	36.9%	956	63.1%	588	61.5%	1515	72.6%	573	27.4%	2088
Cameroon	274	22.9%	923	77.1%	794	86.0%	1197	90.0%	133	10.0%	1330
Rwanda	231	38.1%	376	61.9%	170	45.2%	607	68.4%	280	31.6%	887
South Africa	13,264	48.2%	14,255	51.8%	1789	12.5%	27,519	98.3%	487	1.7%	28,006

^a^ http://gamapserver.who.int/gho/interactive_charts/health_financing/atlas.html?indicator=i2

Private health expenditure (including OOPE) is very high in all countries under investigation. It ranges from 41.3% of THE in Ethiopia to 77.1% of THE in Cameroon. OOPE is a high proportion of THE in all countries except South Africa (where it accounts for 6.5% of THE and 12.5% of private expenditure). In the rest of the sample countries, it ranges from 45.2% of private expenditure in Rwanda to 95.7% the same in Nigeria. OOPE is more than the government expenditure on health in Egypt, Nigeria, DRC and Cameroon (Table 2).

Added to this, ODA is very low in Egypt, Nigeria and South Africa as compared to the ODA in Ethiopia, DRC and Rwanda. It contributes to more than a quarter of the overall health finance (a factor of THE plus ODA) in Ethiopia, DRC and Rwanda. Its contribution is more than that of the government expenditure on health in DRC and Rwanda, and more than private expenditure in Ethiopia (Table 2).

Using the current patterns of financing for health in the case study countries, we have calculated the proportional increases required from each source of finance if universal coverage of HCV treatment is to be realised. National and global strategies may vary the potential increases for each source.

### Required increase in health expenditure by different sources toward universal HCV treatment

In order to achieve the target of HCV elimination, we see in Table 3 that a range of increases would be required by any of the various component sources of financing, even at the current lowest generic drug prices available in 2016 in each country (taken as a baseline for calculations). If drawn from THE alone, expenditures would have to increase by 2% in South Africa and 92% in Cameroon. Additionally, if considering THE as a percentage of GDP, increases would have to be from the present 4.1% of GDP to 7.9% in Cameroon, and from 7.5% to 11.9% in Rwanda. In comparison, the DRC would need to increase current THE as percentage of GDP from 4.3% to 4.5%, and for South Africa this increase would be from 8.8% to 9.0%.

**Table 3:** Required increase in health expenditure by different sources to provide HCV treatment

Country	Total price of Generic	Increase in THE as % of GDP	Increase in THE	Increase in GovtE	Increase in PrvtE	Increase in OOPE	Increase in ODA	Increase in THE plus ODA
Egypt	$5681.3	5.6%–7.3%	31%	80%	50%	55%	2390%	30%
Ethiopia	$507.0	4.9%–5.7%	17%	29%	41%	52%	40%	12%
Nigeria	$6086.3	3.7%–5.0%	34%	136%	46%	48%	510%	32%
DRC	$82.5	4.3%–4.5%	5%	15%	9%	14%	14%	4%
Cameroon	$1104.8	4.1%–7.9%	92%	403%	120%	139%	831%	83%
Rwanda	$356.3	7.5%–11.9%	59%	154%	95%	210%	127%	40%
South Africa	$474.8	8.8%–9.0%	2%	4%	3%	27%	98%	2%

If governments alone were to bear the costs of universal treatment coverage, then the required additional expenditure from present rates would range from a 4% increase in South Africa to a staggering 403% in Cameroon. If drawn exclusively from private expenditure on health, then universal coverage for HCV elimination would require an additional 3% increase in South Africa from present rates to 120% in Cameroon.

If donors were the agents willing to bear the burden of elimination and cover the costs of universal HCV treatment, then the required increase of ODA would vary considerably, from a 14% increase in present ODA for health in DRC, an 831% increase in Cameroon, and a 2390% in Egypt. In general, universal treatment coverage would require additional financing from present rates of THE plus ODA, ranging from 2% in South Africa to 83% in Cameroon.

These expenditures would, however, all be relatively ‘once off’ given the high efficacy of the DAAs and the fact that treatment would prevent onward transmission and new infections, while moving the infected population toward clearing the virus. However, even so, such cost are patently unrealistic for the vast majority of people paying out of pocket and for the majority of governments with limited resources of political will to invest substantially in a public health approach to disease elimination.

### Income inequality, out-of-pocket expenditure, per capita health expenditure and cost of DAA per patient

Table 4 shows median per-capita income, OOPE, per capita health expenditure and cost of HCV treatment per patient in the seven countries, with Gini-coefficients ranging from 30.8 in Egypt to 63.1 in South Africa. The median annual per capita income in these countries is variable and ranges from $308 in DRC to $1217 in South Africa.

**Table 4:** Cost of HCV treatment as percentage of median income and per capita health expenditure in seven countries in Africa

Country	Gini-coefficient^a^	Medina income per capita	Per capita HE^b^	OOPE^b^	Cost of DAA for 12-weeks	Cost of DAA as % of median income	Cost of DAA as % of per capita HE
Egypt	30.8	$623	117.8	55.7%	$684	110%	581%
Ethiopia	33.6	$350	26.7	32.3%	$750	214%	2809%
Nigeria	48.8	$493	117.5	71.7%	$750	152%	638%
DRC	44.4	$308	19.1	38.8%	$750	244%	3927%
Cameroon	38.9	$403	58.7	66.3%	$750	186%	1278%
Rwanda	50.8	$235	52.5	28.0%	$750	319%	1429%
South Africa	63.1	$1217	570.2	6.5%	$750	62%	132%

^a^http://hdr.undp.org/en/content/income-gini-coefficient

^b^ http://gamapserver.who.int/gho/interactive_charts/health_financing/atlas.html?indicator=i2

A high proportion of current health care costs are borne by the patient as OOPE in most of these countries, with the exception of South Africa (Table 4). OOPE is as high as 71.7%, 66.3% and 55.7% of THE in Nigeria, Cameroon and Egypt respectively. Where income inequality is high in countries with high proportions of OOPE, the costs of health care are therefore proportionately more burdensome on the lower income quintiles. The cost of DAAs when represented as percentage of per capita health expenditure is also very high and variable across countries ranging from 132% in South Africa to 3927% in DRC (Table 4).

**Fig. 1:**
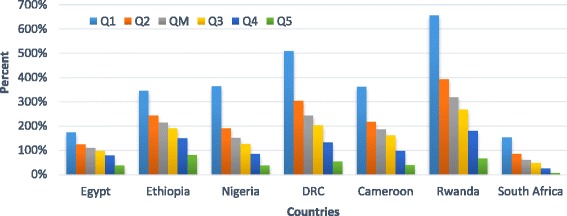
Price of HCV treatment compared to annual income by different wealth quintiles in seven African countries

The price of DAAs for 12-weeks per HCV patient is more than the median income per capita in all countries except South Africa. It ranges from 62% of median annual income in South Africa to 319% in Rwanda, making out-of-pocket payment for HCV treatment completely unrealistic for those in the lower economic quintiles. In countries where the Gini-coefficient is high, this effect is magnified: for example, in Egypt, the cost of the locally produced generic DAA for 12-weeks is estimated to be only 38% of the annual income for a person in the highest wealth quintile and 175% of annual income for a person in the first wealth quintile. In South Africa, the cost of the 12-weeks HCV treatment is estimated to be only 8% of the annual income for a person in the highest wealth quintile, but 154% of annual income for a person in the lowest wealth quintile (Fig. 1).

In Nigeria and Cameroon, the cost of the same HCV treatment is more than the annual income of people in the first, second and third wealth quintiles. In Ethiopia, DRC and Rwanda, the cost of the 12-weeks HCV treatment is more than the annual income of people in all wealth quintiles except those in the fifth quintile (Fig. 1). The required out of pocket spending on DAAs would constitute catastrophic health expenditure by almost any reasonable calculation.

## Discussion

The results of the analysis above clearly challenge expectations of the elimination of HCV by 2030. Achieving such a goal would require increases in expenditure that are either too burdensome to governments or potentially so burdensome to individuals and families that access to these drugs would require, in the worst case scenarios, more than one-year income (with debt therefore being incurred for treatment), clearly leading to catastrophic expenditure. The findings of our study are in line with the recent publication in Lancet that access to quality DAAs and their affordability remain a barrier to achieving universal health coverage of those living with HCV [16].

The major contribution of private health expenditure (OOPE) in all of the countries under investigation, except South Africa, indicates that the financial burden of DAAs would likely fall on individuals. As we have also shown, income inequality combined with OOPE would be, even with the lowest price of treatment available in all case studies countries, out of the range of most of the wealth quintiles. As acknowledged in the 2014 WHO report on health financing, such payments represent a barrier to access to medicines and services, place substantial and often catastrophic burden on individuals and their families, and also have proportionately regressive effects on the poor and the lowest wealth quintiles [17]. This reality of contemporary access to medicines financing has clearly been recognized as a product of unfair or inadequate financing arrangements and risk pooling for health at national level. There needs to be a major impetus towards increased levels of government expenditure as percent of GDP in most LMICs. It is notable that the African countries under consideration are all signatories of the 2001 Abuja declaration on commitment to allocate 15% of the GDP on health. However, as Table 2 indicates, none of the case study countries has achieved this target [18].

The reality for those infected with HCV in the case study countries is that neither their levels of personal income nor government health expenditure will substantially facilitate universal HCV treatment coverage, with countries such Egypt being relatively unique in grounding national commitment to disease elimination on lower priced generic availability. For most in the countries under study this leaves, in essence, only one option: an increase in overseas development assistance for health and there is presently little sign of this having any traction in the donor community.

Universal coverage of HCV infected people at even current lowest generic prices seems in most countries to be unlikely. Indeed,even in the most successful cases of scaling up of ODA for health in recent decades—the global efforts to finance access to antiretrovirals (ARVs) for HIV—coverage has only recently reached 54% of those living with HIV, and that being with the welcomed and aggressive entry of generics into the international market.

While the demand side of HCV treatment is very clear, the supply side is very complex and comparisons with what happened to HIV treatment are illuminating. As with early ARVs, Gilead’s DAAs initially have been set at prices far too high for those living with HCV in almost all countries. Gilead has been criticised internationally for gouging, with the company facing sustained opposition from right to health advocates and patient groups who have argued that there is a moral obligation to treat those facing morbidity and mortality flowing from HCV infection irrespective of the ability to pay [19]. In the context of both HIV and currently HCV, the response by originator firms, in general, and Gilead, in particular, to this opposition has been framed in terms of altruism and corporate social responsibility in discounting prices for certain national markets. These discourses are evident in the framing of voluntary licensing and tiered pricing by originator companies. As our analysis has indicated, even the prices of those generic drugs closest to the marginal cost of production can still exclude poor people from accessing these life-saving drugs in the absence of financing interventions that facilitate equitable access.

Gilead’s current strategy effectively positions it to capture policy, regulation, and political will that maximizes its present market position and profit. If Gilead did not provide tiered-pricing and voluntary licencing for generic production of DAAs, it would face greater opposition and political challenges against its patent monopoly and preponderant international market position. In the case of HIV antiretroviral patent holding firms of the early 2000s [20], their patents were eventually successfully challenged and generics entered the market permitting the scaling up of HIV programs. Even so these firms continued to make profits from their patent and branded HIV drugs in core and other MIC markets [21].

With the above as necessary context, we provide four recommendations that might direct thinking on how to promote wider access to DAAs and progress towards universal coverage: (1) compulsory licensing and patent opposition; (2) political commitment and increased government expenditure; (3) global solidarity and global health financing mechanism; and (4) advocacy by civil society and people living with HCV. We strongly believe that elimination of HCV as a major public health concern is possible if there is political commitment, financial will, global solidarity and civil society activism to expand access to DAAs [15].

### Production and use of generic medicines through compulsory licensing and patent opposition

Compulsory licensing allows any country to authorize a national body to produce or import generic forms of a drug patented in the country. A company can thus produce the patented product or use the patented process without the consent of the patent holder. This would also provide an allowance for export to countries which are unable to manufacture these drugs themselves [22]. The resolution on hepatitis by member states of WHO also urges countries to have national legislative mechanisms for the use of the flexibilities contained in the TRIPS [23].

Therefore, countries with the capacity to manufacture HCV drugs need to enact CL for HCV as it has been the case for HIV and cancer drugs [24]. There are previous experiences from countries, such as Brazil [25], India [26], and Thailand [27], which have issued CLs. These have increased countries’ leverage in negotiating better prices with pharmaceutical companies. This could also be a very good model by which countries could issue CL to roll out generic versions of HCV drugs toward universal coverage.

Patent opposition has clearly shown results in terms of reducing the price of ART [28]. Generic competition has been an effective way to reduce prices for medicines. Each sovereign country, according to WTO, has the right to amend its national patent law to suit its needs. In Brazil, the patent application for tenofovir was rejected by the patent office; and, since 2011, generic version of the medicine has been available, with an estimated savings equivalent to 47% in comparison with Gilead’s price [29].

Argentina has allowed a local company to produce generic sofosbuvir [30]. Morocco, the primary patents for DAAs were not filed, consents for local production and importation of generic products [31]. In Pakistan, the primary patents on sofosbuvir are not filed, local companies have produced generic drugs which has resulted in prices of $45 for a 12-weeks supply of sofosbuvir [32]. Local production of generics in these countries has resulted in lower prices [33].

### Political commitment and increased government expenditure

The countries in this study earmarked only 3.7%–7.7% of their GDP to health, and OOPE in these countries is as high as 71%. Committed and strong leadership, which is willing to improve health financing, able to negotiate for price reduction, and keep to strengthen partnership for advocacy and resource mobilization, is needed to reduce the burden of DAAs on patients and countries.

In Egypt, the price of sofosbuvir for all patients with HCV is equivalent to 48% of the THE and 150% of the government budget for health. In spite of this, Egypt is expanding access to DAAs due to high level of political commitment, effective price negotiations, and local production [34]. Almost 88% of treated patients (close to 700,000) were sponsored by the government whereas only 12% of patients paid OOP [35].

This is a demonstration that it is feasible to increase access to DAAs if governments are committed to the health of their citizens providing resources are available and can be mobilized to that end. It is important that governments increase their health budget to at least 15% of their GDPs as per the Abuja declaration [36]. Governments should also be committed to establish or strengthen their social health insurance system as it is very weak or non-existent in these countries [37].

### Global health financing mechanism for HCV

The Global Fund to fight AIDS, tuberculosis and malaria (Global Fund) has supported many countries to increase access to medicines for AIDS, TB and Malaria [38]. There is however no Global Fund for HCV to purchase drugs for LMICs. Based on the lessons from HIV, countries such as Ethiopia, DRC, Cameroon, Nigeria and Rwanda, will face huge constraints to provide DAAs. It is thus vital that the mechanism that facilitate the large scale expansion of medicines for AIDS, TB and Malaria, will be introduced to support countries struggling to provide DAAs for people living with HCV.

It is also important that the lessons from the Global Fund can be used to mobilize and pool the development assistance for HCV, and establish a Global Fund for HCV. The World Health Organization has already spearheaded the global movement against HCV and integrated HCV with HIV control. The Global Fund should also be mandated and resourced to move in this direction and given additional capacity to start financing the DAAs in LMICs. This may also create an opportunity to increase the capacity to procure drugs at national and multi-national levels, and expand access to them in LMICs. This mechanism may also be integrated with the existing Global Fund mechanism and expanded to include other health problems including HCV.

### Civil society movements

Civil society movements have helped the massive scale up of antiretroviral treatment in many countries [39]. These movements have also led to expansion of DAAs for HCV. In Brazil, civil societies advocated for prices reductions and increased government commitments which has led to provision of the treatment free of charge [40]. In other countries, such as Thailand, Argentina, and Egypt, civil societies have hard-pressed for increased access to DAAs [41]. Countries, such as Georgia, Ukraine and Rwanda, are providing DAAs with a support from non-governmental organizations with a lot of advocacy from civil societies [42].

This study has both strengths and limitations. The strength is that it is a multi-country study including both MICs and LICs where the burden of HCV is very high. This will improve the application of the knowledge to other similar contexts. The limitation of the study is that it is based on secondary data that may lack quality. To minimize this we have used the same data sources consistently and triangulated different data sources to confirm data provided. We have used conservative estimates such as lowest prices for DAAs and12-weeks regimen; these may underestimate the burden of HCV treatment. Finally, this study focuses on pricing, though increasing access to DAAs also requires other aspects, including strong health system components such as service delivery and human resources, which need to be dealt if countries are to achieve the goal of ending HCV.

## Conclusion

The present aspirations, for increasing access to DAAs towards the elimination of HCV, are facing challenges that would require increases in expenditure that are either too burdensome to governments or completely catastrophic for lower income individuals and families. This is mainly due to pricing and licensing strategy that acts as a barrier to would-be generic entry which could introduce further competition and drive prices down closer to the marginal cost of production. Countries, therefore, need to implement flexibilities including compulsory licensing and patent opposition, and manufacture generic drugs which can be made available to people living with HCV at much lower prices. Moreover, elimination of HCV as a major public health concern requires political commitment, financial will, global solidarity and civil society activism to expand access to DAAs in LMICs.

## Abbreviations

DAAs: Direct Acting Antivirals
DRC: Democratic Republic of Congo
GDP: Gross Domestic Product
HCV: Hepatitis C Virus
HICs: High income countries
HICs: High income countries,
LICs: Low-income countries
LMICs: Low- and middle-income countries
MICs: Middle income countries,
ODA: Overseas development assistance
OOPE: Out-of-pocket-expenditure
THE: Total health expenditure
